# Supplementary material for: A prospective, multicentre study evaluating safety and efficacy of a fixed dose combination of Remogliflozin etabonate, Vildagliptin, and Metformin in Indian patients with type 2 diabetes mellitus (Triad-RMV)
Source: Clin Diabetes Endocrinol. 2024 Dec 18;10:49. doi: 10.1186/s40842-024-00210-8 (PMC11654189; doi:10.1186/s40842-024-00210-8)
Supplement: Supplementary file 1 — Supplementary Material 1 [file 40842_2024_210_MOESM1_ESM.pdf]

Title

A Prospective, Multicentre Study Evaluating Safety and Efficacy of a Fixed Dose Combination of Remogliflozin Etabonate, Vildagliptin, and Metformin in Indian Patients with Type 2 Diabetes Mellitus (Triad-RMV)

Authors Information

Prabhat K Agarwal[1], Divendu Bhushan[2], Amit Bhate[3], Sunil Naik[4], Shailesh Adwani[5], J S Kushwaha[6], Sumit Bhushan[7*], Abhishek Mane[8], Rujuta Gadkari[9], Sanjay Choudhari[10], Saiprasad Patil[11], Hanmant Barkate[12]

[1]S.N. Medical college, Agra, India (ppagrawal120@gmail.com)

[2]AIIMS, Patna, Bihar, India (drdivendubhushan@gmail.com)

[3]Jeevan Rekha Hospital, Belagavi, India (dr.amitsureshbhate@gmail.com)

[4]Govt. Medical college, Srikakulam, India (drsunilnaikggh@gmail.com)

[5]PDEA'S Ayurved & multispecialty hospital, Pune, India (sradwani@gmail.com)

[6]Prakhar hospital, Kanpur, India (dr.jskushwahacr@gmail.com)

[7*] Glenmark Pharmaceuticals, Mumbai, India (sumit.bhushan@glenmarkpharma.com)

[8,9,10,11,12]Glenmark Pharmaceuticals, Mumbai, India (abhishek.mane1@glenmarkpharma.com, rujuta.gadkari@glenmarkpharma.com, sanjay.choudhari@glenmarkpharma.com, Saiprasad.patil@glenmarkpharma.com, Hanmant.barkate@glenmarkpharma.com)

*Corresponding author

Abstract

Aims: The ICMR INDIAB-17 study revealed a diabetes prevalence of 11.4% in India, emphasizing the need for effective treatment for glycemic control. A Phase IV study was conducted to evaluate the safety and efficacy of a Fixed Dose Combination (FDC) of Remogliflozin, Metformin and Vildagliptin (RMV) in Type 2 Diabetes Mellitus (T2DM) patients uncontrolled on Metformin plus SGLT2 inhibitor or Metformin plus DPP4 inhibitor dual therapy.

Methods: A total of 215 patients (mean age: 46.4 years; 64% male, 36% female) were enrolled across multiple centers in India. The study population included patients with a baseline HbA1c ≥ 8% at the time of screening. The primary objective was to assess safety based on treatment-emergent adverse events (TEAEs), while the secondary aim was to evaluate effectiveness in terms of glycemic (HbA1c, fasting plasma glucose, postprandial glucose) and extra-glycemic measures (renal and lipid parameters). Statistical analysis was conducted using paired t-tests and the Wilcoxon signed-rank test for within-group comparisons, and the Bonferroni correction was applied to adjust for multiple comparisons. Effectiveness was evaluated at baseline, week 12, and week 24.

Results: ~~215 enrolled patients~~The study demonstrated statistically significant ~~improvements~~ reductions in mean HbA1c levels from baseline to both week 12 and week 24 ($p<0.00001$). At 24, weeks, ~~Approximately~~ 45.1% of patients achieved target HbA1c levels of ≤7%~~ at 24 weeks,~~. ~~with s~~Significant reduction~~s~~ was also observed in fasting plasma glucose (FPG) and postprandial glucose (PPG) levels~~ observed~~. Renal parameters remained stable or improved, and lipid profile parameters, including LDL-C and triglycerides, ~~also~~ showed ~~improvement~~favorable changes. ~~Weight reduction was noted, and a~~Adverse events of special interest, including hypoglycemia and urinary tract infections, were reported in 4.7% of patients, ~~without an~~with no~~y~~ serious adverse event~~s~~ ~~observed~~recorded.

Conclusions: ~~There was significant improvement in HbA1c, FPG and PPG from baseline to week 12 and week 24. Hence,~~The twice daily triple FDC of RMV was ~~found to be~~ well tolerated, safe and effective in patients with Type 2 Diabetes Mellitus uncontrolled on dual drug therapy of Metformin plus SGLT2i or Metformin plus DPP4i. The treatment led to significant improvements in glycemic control and other metabolic parameters over 24 weeks, without compromising renal function or causing serious adverse events.

Trial registration: CTRI, CTRI/2022/05/042581. Registered 17 May 2022,
https://ctri.nic.in/Clinicaltrials/rmaindet.php?trialid=68757&EncHid=36127.16500&modid=1&compid=19

Keywords: Fixed dose combination, Remogliflozin, Vildagliptin, Metformin, Type 2 Diabetes, Indian study

Background

In recent decades, India has shown a rapid rise in the prevalence of Type 2 Diabetes Mellitus (T2DM). Recently published ICMR-INDIAB 17 study reported the prevalence of diabetes and prediabetes in India as 11.4% and 15.3% respectively (1). T2DM is a progressive metabolic disorder where the risks of microvascular and macrovascular complications, as well as mortality, are closely linked to hyperglycaemia. The primary objective of diabetes treatment is to achieve and maintain optimal glycemic control to prevent these complications (2). Also, when it comes to effective T2DM management, addressing the associated comorbidities is equally important for improving the patient benefit and disease outcomes. There are numerous therapies currently available to help T2DM patients reach their glycemic goals. Most clinical guidelines recommend Metformin as a first line medication for T2DM. Metformin is preferred due to its efficacy, low cost, and good safety profile. However, many patients cannot achieve consistent glycemic control with Metformin alone and require treatment intensification with multiple drug regimens as the disease progresses.

T2DM is associated with a complex multi-system pathophysiology, which requires a multi-faceted treatment approach. Combinations with different anti-diabetic drug classes that have multiple mechanism of action have potential advantages like early, greater, and sustained reduction in HbA1c, improved patient compliance due to reduced pill burden and reduced clinical inertia (3,4). Sulfonylureas (SUs) and dipeptidyl peptidase-4 inhibitors (DPP-4i) are the most commonly used second line agents as an add-on to Metformin monotherapy.

Drug adherence is another factor that gets better on using fixed drug combinations or FDCs. However, such FDCs should be justified with a strong rationale based on factors such as different but complementary mechanisms, compatible pharmacokinetic profile, and good safety profile. In the year 2020, USFDA has for the first-time approved triple combination oral therapy consisting of a SGLT2 inhibitor Empagliflozin, the DPP-IV inhibitor Linagliptin and Metformin hydrochloride extended release (5) (6).

Previously published evidence has pointed out that the combination of SGLT2i+DPP4i with or without Metformin is associated with superior glycaemic control. This highlights the complementary anti-diabetic mechanism of action of these two classes of oral antidiabetic drugs. Their combination has also shown a significant reduction in weight and a risk of hypoglycaemia (7).

Studies have also shown that combination therapies of SGLT2 inhibitors with other agents like DPP4I and Metformin have been well tolerated and the frequency of Adverse Events (AEs) is comparatively greater for monotherapy (79.1%) v/s combination therapy (72.4%) (8). Another advantage of using this combination is reduced incidence of genital tract infections as compared to SGLT2i therapy alone (9).

Treatment failures have been reported for dual combination of Metformin along with SUs and DPP-4i (10). Thus, for patients inadequately controlled on dual combination agents, a triple combination therapy with sodium–glucose cotransporter-2 inhibitors (SGLT-2i), DPP4i and Metformin has multiple benefits. The mechanisms of action of DPP4i and SGLT2i are complementary to that of Metformin with low risk of hypoglycaemia (2,11).

Recent guidelines like American Diabetes Association (ADA) *Standards of Care in Diabetes–2024* recommends early combination therapy incorporating high-glycemic-efficacy therapies (e.g. combination oral agents) in people presenting with A1c levels 1.5–2.0% above goal (12). According to the American Association of Clinical Endocrinology (AACE) Comprehensive Type 2 Diabetes Management Algorithm – 2023 Update, if the initial A1c is >9% or 1.5% above goal, then a combination of 2 or 3 antihyperglycemic agents should be initiated (13).

Therefore, in uncontrolled T2DM patients, early initiation of triple combination can be considered as an efficacious option.

The combination of Remogliflozin, Vildagliptin, and Metformin was developed as a promising pharmacotherapeutic option, representing the first approval of its kind in India. Currently available SGLT2 inhibitors have been extensively studied in Caucasians, but their applicability to Asian and Indian populations, characterized by genetic, BMI, dietary, and diabetes differences, remains uncertain. Despite India's high diabetes prevalence, no SGLT2 inhibitor has been specifically studied in its population. Remogliflozin, unique for its twice-daily dosing, demonstrated efficacy and safety in Indian population. Its combination with Vildagliptin and Metformin offers effective glycemic control for uncontrolled Indian T2DM patients, considering their unique phenotype and dietary habits.

Therefore, this phase IV clinical trial was planned to assess the safety and efficacy of this combination in Indian patients with Type 2 Diabetes Mellitus.

Material and Methods

Study Design

TRIAD-RMV was a prospective, multi-centre, baseline controlled, single arm, interventional study to assess the safety and efficacy of triple drug FDC of Remogliflozin, Metformin and Vildagliptin in T2DM patients. The trial was conducted at 7 sites across India. The research outlined in this study adheres to the study protocol, the New Drugs and Clinical Trials Rules 2019 issued by Indian Government, ethical principles derived from the Declaration of Helsinki, International Council for Harmonisation (ICH) Good Clinical Practice (GCP) guidelines, and all relevant local regulatory standards. Informed consent was obtained from all the study subjects who took part in the trial. Ethics Committee approval was obtained from all respective sites and study was registered in CTRI (CTRI/2022/05/042581).

Subjects

The study enrolled Indian patients between age group 18-65 years of either gender, diagnosed with T2DM for >6 months and who were uncontrolled (HbA1c ≥ 8% at time of screening) on dual drug regimen of Metformin+DPP4i or Metformin+SGLT2i for last 12 weeks before screening. The daily Metformin dose was either 1000mg or 2000mg. Patients were ineligible if they reported a history of hypersensitivity or contraindication to the study drug combination, moderate to severe hepatic, cardiac, neurologic or neoplastic disorder, eGFR <60 mL/min/1.73 m^2 at the time of initiation of Remogliflozin Etabonate (RE) therapy (eGFR of <45 L/min/1.73m^2), pancreatitis or pancreatic surgery, any malignancy, diabetic ketoacidosis or any acute/chronic systemic infections. Patients with Type 1 Diabetes, recurrent urogenital infections, pregnant or breastfeeding women and individuals at risk for volume depletion as judged by the investigator were also excluded.

Study Interventions

Patients enrolled in the study were dispensed with the study medication of FDC of Remogliflozin etabonate 100 mg, Vildagliptin 50 mg and Metformin 500 mg/ 1000 mg tablets given orally twice daily (Approx. 9 AM at breakfast and 9 PM at dinner). The duration of the study was 24 weeks. The patients were provided with glucometers for Self-Monitoring of Blood glucose (SMBG) and subject diary cards also provided to capture all solicited and unsolicited adverse events and noting down the consumption of investigational product. The subjects were informed about the solicited adverse events and were trained on performing SMBG and data entry in the

subject diary. The subjects were informed to visit the facility after every 3 months for physical follow-up visit or any time during the interval in case of any adverse events. Telephonic follow-ups were performed at 4, 8 and 18 weeks after baseline visit for active safety monitoring and assessment of treatment compliance. On every follow-up visit, a complete clinical and laboratory assessment was performed. Subject diary review was performed at each physical visit for active safety monitoring and assessment of treatment compliance.

Endpoints

The efficacy endpoints included assessing the average change in HbA1c levels from baseline to week 24. Other efficacy measures included changes in Fasting Plasma Glucose (FPG), Post-Prandial Plasma Glucose (PPG) levels, body weight, waist circumference, and blood pressure at Weeks 12 and 24. The proportion of patients achieving target HbA1c levels of ≤7% at Weeks 12 and 24 was also monitored, along with tracking the number of patients needing additional medications by the 12- and 24-week marks. Furthermore, alterations in Triglycerides (TG), Total Cholesterol (TC), Low-Density Lipoproteins (LDL), and High-Density Lipoproteins (HDL) from baseline to week 24 were documented. Safety assessments involved recording the occurrence of treatment-emergent adverse events (TEAEs) of special interest as evaluated by physicians over the 24-week period.

Statistical Analysis

~~The data was analysed using appropriate statistical techniques.~~ To evaluate the significant improvement in glycemic parameters (e.g., HbA1c, fasting plasma glucose) from baseline to 24 weeks, the Wilcoxon signed-rank test was utilized. This non-parametric test is appropriate for comparing paired data across different visits, as it does not assume a normal distribution. It is well suited for changes in repeated measurements within the same participants (e.g., baseline, interim, and final assessments). With multiple visits (e.g., baseline, 12 weeks, and 24 weeks), comparisons were made across time points to assess the improvement trajectory. To address the risk of type I error from multiple comparisons, the Bonferroni correction was applied, ensuring a robust significance threshold. All safety and efficacy endpoints were analysed using Modified Intent-to-Treat (mITT) set and safety set respectively. The analyses of the primary and secondary endpoints were performed in Per Protocol (PP) and ITT (Intention to Treat) populations. mITT population included all enrolled subjects who received at least 1 dose of study drug, who had a non-missing baseline measurement and at least 1 post-baseline efficacy measurement for primary efficacy variable. Per protocol population included all enrolled subjects receiving at least 1 dose of study drug, who have non-missing baseline measurement and at least 1 post baseline measurement of primary efficacy variable, completed the study, and did not have any major protocol deviations. Safety population included all enrolled subjects who received at least 1 dose of study drug.

Results

Among the 254 subjects screened, 39 were deemed screen failures. A total of 215 adult Indians (age ≥18 years, ≤65 years) of either gender, diagnosed with T2DM for more than 6 months, were enrolled in the study. All 215 subjects completed the study as per the protocol. A summary of key baseline demographic characteristics for the study subjects is mentioned in **Table 1.**

Of the 215 subjects, 77 (35.8%) were female, and 138 (64.2%) were male, with a mean age of 46.4 years. The weight and waist circumference of the subjects at baseline were 67.7 kg and 85.6 cm, respectively.

A total of 81 subjects (37.67%) took concomitant medications during the study period. Most commonly observed comorbidity was hypertension, reported in 39 subjects (18.14%) followed by neuropathy (9.77%), dyslipidaemia (8.37%), hypothyroidism (3.26%) and benign prostatic hyperplasia (BPH) (0.47%).

Safety Results

Overall, a total of 53 (24.7%) subjects in the study population (N=215) had 70 adverse events (AEs) mentioned in **Table 2.** Of the 70 AEs, 46 were solicited adverse events and 24 were unsolicited adverse events. Also of the 70 AEs, 10 Adverse Events of Special Interest (AESIs) were reported. The outcome of all 70 AEs was completely resolved.

Adverse Events reported during the study period were back pain, body ache, cold, cough, diarrhoea, dizziness, fatigue, fever, GI distress, headache, hypoglycaemia, myalgia, nausea, runny nose, stomach-ache, Urinary Tract Infection (UTI), vomiting and genital infections (balanitis and vulvovaginitis).

Efficacy Results

The mean HbA1c (%) of the subjects was 9.2 ± 0.9 at baseline which improved to 7.9 ± 1.0 at week 12 and 7.2 ± 1.1 at week 24 respectively. The mean FBG (mg/dl) was 173.2 ± 37.1 at baseline and improved to 147.6 ± 31.9 at week 12 and 135.8 ± 28.7 at week 24. The mean PPG (mg/dl) was 258.9 ± 46.3 at baseline and improved to 218.1 ± 42.8 at week 12 and 202.7 ± 39.7 at week 24. Similarly, the proportion of patients achieving target glycemic control of HbA1c ≤ 7 % was 14.4% at week 12 and 45.1% at week 24 **(Figure 1: A, B, C D)**.

There was also significant reduction in Blood Pressure, weight, and waist circumference of the subjects over 24 weeks of treatment. Systolic blood pressure (mmHg) from baseline value of 126.2 ± 5.7 improved to 124.6 ± 5.1 at week 24. The diastolic blood pressure from baseline value of 79.1 ± 5.2 improved to 77.8 ± 5.4 at week 24. The weight (Kg) of the subjects from baseline value of 67.7 ± 7.8 reduced to 65.0 ± 8.0 at week 24 and the waist circumference (cm) from baseline value of 85.6 ± 7.4 reduced to 83.7 ± 7.3 at week 24.

Renal parameters of all subjects were well maintained or improved throughout the study duration. The serum creatinine of the subjects was 0.95 ± 0.18 at baseline and 0.94 ± 0.20 at week 12 and 0.93 ± 0.20 at week 24 respectively. The eGFR of the subjects was 84.88 ± 17.42 at baseline and 84.60 ± 19.43 at week 12 and 87.05 ± 17.39 at week 24 respectively **(Figure 2: A, B)**.

The mean of TC (mg/dL) from baseline value of 188.5 ± 32.7 reduced to 179.9 ± 31.7 at week 24. LDL (mg/dL) was 116.5 ± 32.5 at baseline which reduced to 106.2 ± 31.8 at week 24. TG (mg/dL) from baseline value of 156.3 ± 55.0 reduced to 148.6 ± 55.8 at week 24 **(Figure 3: A, B, C).**

The values for Liver Function Tests were well maintained throughout the study duration. The Alanine Transaminase (ALT) of subjects was 38.08 ± 12.48 at baseline and 39.41 ± 10.89 at week 12 and 38.49 ± 16.12 at week 24 respectively. The Aspartate Transaminase (AST) of subjects was 38.14 ± 12.81 at baseline and 38.34 ± 10.99 at week 12 and 37.34 ± 10.99 at week 24 respectively. The Total Bilirubin of subjects was 0.73 ± 0.22 at baseline and 0.74 ± 0.23 at week 12 and 0.72 ± 0.24 at week 24 respectively **(Figure 4: A, B, C).**

Discussion

For most individuals with T2DM, initiating metformin is advised as the first-line therapy to enhance glycemic regulation and mitigate the risk of diabetes-related complications. If metformin proves inadequate in sustaining glycemic control, the addition of antihyperglycemic medications with mechanisms of action that complement metformin should be contemplated. The multifactorial 'ominous octet' delineates the central pathophysiology of

diabetes, with different classes of anti-diabetic agents targeting distinct components of this octet. This necessitates the addition of drugs that complementarily target established defects in T2DM (14). In this regard, multiple single-dose agents formulated as a single-dose form called fixed-dose combinations (FDCs) have been evaluated for their safety, efficacy, and tolerability. FDCs, whether double or triple, enhance patient adherence, lower costs, and deliver effective glycemic control. Consequently, they play a pivotal role in the management of type 2 diabetes mellitus (T2DM). Initiating combination therapy early establishes a favorable legacy effect, contributing to the enhancement of patients' glycemic profiles, with minimal escalation in the occurrence of side effects (15).

DPP-4 inhibitors and SGLT2 inhibitors are acknowledged as one of the best supplementary choices for incorporating into dual or triple therapy alongside metformin. DPP-4 inhibitors lower blood glucose levels by inhibiting GLP-1 degradation, stimulating insulin secretion, and curbing glucagon secretion. SGLT2 inhibitors represent the latest class of oral antidiabetes medications approved by both the U.S. Food and Drug Administration and the European Medicines Agency. SGLT2 inhibitors function independently of insulin, thus posing a low risk of hypoglycemia. Additionally, they offer the added advantage of weight reduction due to caloric loss in the urine of about 200–340 kcal/day (16).

A triple-drug regimen integrating DPP4i, Metformin, and SGLT2i can efficiently address diverse aspects of T2DM, including insulin resistance, β-cell dysfunction, and glucose reabsorption. Various studies have shown efficacy of triple FDCs in T2DM management.

While the estimated count of FDCs available in India exceeds 6000, numerous reports, studies, and editorials have highlighted insufficient data as the primary obstacle in establishing the safety and efficacy of these FDCs (15). Moreover, current research on SGLT2 inhibitors and its combinations primarily focuses on Caucasians, with minimal representation from Asian and Indian populations. However, Indians differ from Caucasians in genetics, Body Mass Index (BMI), dietary habits, diabetes nature, and responses to glucose-lowering medications. Consequently, findings from studies in these populations may not be directly applicable to Indian groups (17).

Remogliflozin etabonate, a new oral prodrug of remogliflozin, in its Phase 3 trial in Indian population has demonstrated good efficacy and safety profile in a head-to-head comparison against Dapagliflozin (17). This is the only SGLT-2i in the market with twice daily dosing making it suitable for Indian patients with a distinctive phenotype and a high carbohydrate intake in the form of three daily meals. The combination of Remogliflozin and Vildagliptin with Metformin presents the optimal choice for achieving effective glycemic control in uncontrolled Indian diabetic patients, whether they have comorbidities or not.

Studies have emphasized the effectiveness and safety of this trio in patients with T2DM. An observational study was conducted to assess whether adding the FDC of Remogliflozin and Vildagliptin to existing therapy could enhance glycemic control and have non-glycemic benefits on physical parameters, blood pressure, lipids, and insulin resistance in T2DM patients. The study observed a statistically significant reduction in mean HbA1c at the third and sixth month from baseline however there was no significant effect on body mass index, blood pressure, and lipids (18). Another 16-week randomized, active-controlled, double-blind, phase III study investigated the efficacy and safety of the FDC of Remogliflozin Etabonate and Vildagliptin (RV) versus Empagliflozin and Linagliptin (EL) in T2DM patients. Both groups exhibited comparable significant reductions in Fasting Plasma Glucose, Post-Prandial Plasma Glucose, body weight, and blood pressure, with overall good tolerability. Thus, adding Remogliflozin Etabonate and Vildagliptin to Metformin was shown to be non-inferior to Empagliflozin 25mg + Linagliptin 5mg treatment alongside Metformin (19, 20).

The current prospective, multicentre study showed that initiation of triple drug fixed dose combination (FDC) of Remogliflozin Etabonate, Metformin and Vildagliptin (RMV) given twice daily orally showed significant improvement in HbA1c, FPG and PPG from baseline to week 12 and week 24. These results are on the lines of a study by DeFronzo *et al.* conducted to evaluate the efficacy of combination of empagliflozin/linagliptin as second-line therapy in patients with type 2 diabetes inadequately controlled on Metformin. The reductions in HbA1c with empagliflozin/linagliptin were superior to those with empagliflozin or linagliptin alone as add-on to Metformin (21).

In the current study, the triple combination also yielded additional glycemic benefits, along with significant reductions in blood pressure, lipid parameters, and weight over the 24-week treatment period. There was no observed decline in renal parameters, and both eGFR and serum creatinine values remained well maintained. Moreover, no severe adverse events were reported in the study. These added benefits are comparable to those observed with the triple combination of empagliflozin, linagliptin and Metformin (22).

The study had some limitations such as its single-arm design, short follow-up duration, relatively small sample size, exclusion of patients with certain comorbidities or conditions, and potential confounders like changes in lifestyle or concurrent medications, which could impact the outcomes. Therefore, further studies with longer follow-up durations and larger sample sizes are needed to validate these findings and assess the long-term safety and efficacy of this combination therapy.

Conclusion

There was significant improvement in HbA1c, FPG and PPG from baseline to week 12 and week 24. Hence, twice daily triple FDC of RMV was found to be well tolerated, safe and effective in patients with Type 2 Diabetes Mellitus uncontrolled on dual drug therapy of Metformin plus SGLT2i or Metformin plus DPP4i.

References

1. Anjana RM, Unnikrishnan R, Deepa M, et al. Metabolic non-communicable disease health report of India: the ICMR-INDIAB national cross-sectional study (ICMR-INDIAB-17). Lancet Diabetes Endocrinol. 2023 Jul;11(7):474-489. doi: 10.1016/S2213-8587(23)00119-5.

2. Sahay RK, Giri R, Shembalkar JV, et al. Fixed-Dose Combination of Dapagliflozin + Sitagliptin + Metformin in Patients with Type 2 Diabetes Poorly Controlled with Metformin: Phase 3, Randomized Comparison with Dual Combinations. Adv Ther. 2023 Jul;40(7):3227-3246. doi: 10.1007/s12325-023-02523-z.

3. Zhu NA, Harris SB. Therapeutic Inertia in People With Type 2 Diabetes in Primary Care: A Challenge That Just Won't Go Away. Diabetes Spectr. 2020 Feb;33(1):44-49. doi: 10.2337/ds19-0016.

4. Xie X, Wu C, Hao Y, et al. Benefits and risks of drug combination therapy for diabetes mellitus and its complications: a comprehensive review. Front Endocrinol (Lausanne). 2023 Dec 19;14:1301093. doi: 10.3389/fendo.2023.1301093.

5. John M, Gopinath D, Kalra S. Triple fixed drug combinations in type 2 diabetes. Indian J Endocrinol Metab. 2015 May-Jun;19(3):311-3. doi: 10.4103/2230-8210.152739.

6. FDA approves first triple combination pill for type 2 diabetes. 2020 https://www.healio.com/news/endocrinology/20200128/fda-approves-first-triple-combination-pill-for-type-2-diabetes

7. Min SH, Yoon JH, Moon SJ, et al. Combination of sodium-glucose cotransporter 2 inhibitor and dipeptidyl peptidase-4 inhibitor in type 2 diabetes: a systematic review with meta-analysis. Sci Rep. 2018 Mar 13;8(1):4466. doi: 10.1038/s41598-018-22658-2.

8. Kalra S, Kesavadev J, Chadha M, et al. Sodium-glucose Cotransporter-2 Inhibitors in Combination with Other Glucose-lowering Agents for the Treatment of Type 2 Diabetes Mellitus. Indian J Endocrinol Metab. 2018 Nov-Dec;22(6):827-836. doi: 10.4103/ijem.IJEM_162_17.

9. Fadini GP, Bonora BM, Mayur S, et al. Dipeptidyl peptidase-4 inhibitors moderate the risk of genitourinary tract infections associated with sodium-glucose co-transporter-2 inhibitors. Diabetes Obes Metab. 2018 Mar;20(3):740-744. doi: 10.1111/dom.13130.

10. Lee S, Lee S, Jang IJ, et al. Electronic medical records-based comparison of glycemic control efficacy between sulfonylureas and dipeptidyl peptidase-4 inhibitors added on to metformin monotherapy in patients with type 2 diabetes. Transl Clin Pharmacol. 2020 Dec;28(4):199-207. doi: 10.12793/tcp.2020.28.e21.

11. Handelsman Y, Mathieu C, Del Prato S, et al. Sustained 52-week efficacy and safety of triple therapy with dapagliflozin plus saxagliptin versus dual therapy with sitagliptin added to metformin in patients with uncontrolled type 2 diabetes. Diabetes Obes Metab. 2019 Apr;21(4):883-892. doi: 10.1111/dom.13594.

12. American Diabetes Association Professional Practice Committee. Erratum. 9. Pharmacologic Approaches to Glycemic Treatment: Standards of Care in Diabetes-2024. Diabetes Care 2024;47(Suppl. 1):S158-S178. Diabetes Care. 2024 Jul 1;47(7):1238. doi: 10.2337/dc24-er07a.

13. Samson SL, Vellanki P, Blonde L, et al. American Association of Clinical Endocrinology Consensus Statement: Comprehensive Type 2 Diabetes Management Algorithm - 2023 Update. Endocr Pract. 2023 May;29(5):305-340. doi: 10.1016/j.eprac.2023.02.001.

14. Chadha M, Das AK, Deb P, et al. Expert Opinion: Optimum Clinical Approach to Combination-Use of SGLT2i + DPP4i in the Indian Diabetes Setting. Diabetes Ther. 2022 May;13(5):1097-1114. doi: 10.1007/s13300-022-01219-x.

15. Kalra S, Das AK, Priya G, et al. Fixed-dose combination in management of type 2 diabetes mellitus: Expert opinion from an international panel. J Family Med Prim Care. 2020 Nov 30;9(11):5450-5457. doi: 10.4103/jfmpc.jfmpc_843_20.

16. Matthaei S, Catrinoiu D, Celiński A, et al. Randomized, Double-Blind Trial of Triple Therapy With Saxagliptin Add-on to Dapagliflozin Plus Metformin in Patients With Type 2 Diabetes. Diabetes Care. 2015 Nov;38(11):2018-24. doi: 10.2337/dc15-0811.

17. Dharmalingam M, Aravind SR, Thacker H, et al. Efficacy and Safety of Remogliflozin Etabonate, a New Sodium Glucose Co-Transporter-2 Inhibitor, in Patients with Type 2 Diabetes Mellitus: A

24-Week, Randomized, Double-Blind, Active-Controlled Trial. Drugs. 2020 Apr;80(6):587-600. doi: 10.1007/s40265-020-01285-0.

18. Petchiappan V, Mathew E, Jose J, et al. Use of Fixed-dose Combination Therapy with Remogliflozin and Vildagliptin as an Add-on Drug in Improving the Glycemic Control of Type 2 Diabetes Mellitus: An Observational Study. Journal of Pharmacology and Pharmacotherapeutics. 2023;14(1):72-78. doi:10.1177/0976500X231175219

19. Khaladkar K, Mohan B, Khaladkar K, et al. Efficacy and Safety of a Fixed Dose Combination of Remogliflozin Etabonate and Vildagliptin in Patients with Type-2 Diabetes Mellitus: A Randomized, Active-Controlled, Double-Blind, Phase III Study. J Assoc Physicians India. 2022 Apr;70(4):11-12.

20. Shamanna P, Mohan B, Aravind SR, et al. Efficacy and Safety of a Combination of Remogliflozin Etabonate and Vildagliptin as Fixed Doses in Patients with Type-2 Diabetes Mellitus: A Randomized, Active Controlled, Double-Blind Study. (FOREVER STUDY). Curr Trends Intern Med 2024;Volume 8(1):1-10. DOI: 10.29011/2638-003X.100115

21. DeFronzo RA, Lewin A, Patel S, et al. Combination of empagliflozin and linagliptin as second-line therapy in subjects with type 2 diabetes inadequately controlled on metformin. Diabetes Care. 2015 Mar;38(3):384-93. doi: 10.2337/dc14-2364.

22. HIGHLIGHTS OF PRESCRIBING INFORMATION. 2020 https://www.accessdata.fda.gov/drugsatfda_docs/label/2020/212614s000lbl.pdf

Declaration

Ethics approval and consent to participate: The work presented in this study was in accordance with the study protocol, the New Drugs and Clinical Trials Rules 2019 issued by the Government of India, the ethical principles that have their origin in the Declaration of Helsinki, International Council for Harmonisation (ICH) Good Clinical Practice (GCP), and all applicable local regulatory requirements. Informed consent was obtained from all the study subjects who took part in the trial. Ethic committee approval was obtained from all respective sites and study was registered in CTRI (CTRI/2022/05/042581).

Consent for publication: We, hereby transfer all copyright ownership of the manuscript entitled " A Prospective, Multicentre Study Evaluating Safety and Efficacy of a Fixed Dose Combination of Remogliflozin Etabonate, Vildagliptin, and Metformin in Indian Patients with Type 2 Diabetes Mellitus (Triad-RMV)" to 'Clinical Diabetes and Endocrinology' effective from the date of submission.

We affirm that the manuscript is original, that we are the authors of the manuscript, and that it has not been published previously nor is it under consideration for publication elsewhere. We also affirm that the manuscript does not infringe upon any existing copyright or any other rights of any third party and that we have obtained all necessary permissions for any third-party materials included in the manuscript.

Availability of data and material: The datasets generated and analyzed during the current study are available from the corresponding author.

Competing interests: Corresponding author has received funding from Glenmark Pharmaceuticals Ltd for research and its publication. The other authors declare that they have no competing interests.

Funding: This study was funded by Glenmark Pharmaceuticals Limited. This research did not receive any specific grant from funding agencies in the public, commercial, or not-for-profit sectors.

Authors' contributions: All authors have read and approved the final manuscript and agree to be accountable for all aspects of the work.

Acknowledgements: We would like to extend our thanks to all the institutes and respective investigators and team members for their support. We also appreciate the site management support by Clinical Research Network India, New Delhi, India.
